# A brief psychological intervention to reduce repetition of self-harm in patients admitted to hospital following a suicide attempt: a randomised controlled trial

**DOI:** 10.1016/S2215-0366(17)30129-3

**Published:** 2017-06

**Authors:** Rory C O'Connor, Eamonn Ferguson, Fiona Scott, Roger Smyth, David McDaid, A-La Park, Annette Beautrais, Christopher J Armitage

**Affiliations:** a Suicidal Behaviour Research Laboratory, Institute of Health & Wellbeing, University of Glasgow, Glasgow, UK; b School of Psychology, University of Nottingham, Nottingham, UK; c Department of Psychological Medicine, Royal Infirmary of Edinburgh, Edinburgh, UK; d Personal Social Services Research Unit, Department of Social Policy, London School of Economics, London, UK; e School of Health Sciences, University of Canterbury, Christchurch, New Zealand; f Manchester Centre for Health Psychology, University of Manchester, Manchester, UK

## Abstract

**Background:**

We investigated whether a volitional helpsheet (VHS), a brief psychological intervention, could reduce repeat self-harm in the 6 months following a suicide attempt.

**Methods:**

We did a prospective, single-site, randomised controlled trial. Patients admitted to a hospital in Edinburgh, UK, after a suicide attempt were deemed eligible for the study if they were over the age of 16 years, had a self-reported history of self-harm, were fluent in English, were medically fit to interview, and were not participating in other research studies within the hospital. Eligible patients were randomly assigned (1:1), via web-based randomisation, to receive either VHS plus usual treatment (intervention group) or only treatment as usual (control group). Randomisation was stratified by sex and self-reported past self-harm history. The Information Services Division of the National Health Service (NHS-ISD) staff and those extracting data from medical notes were masked to the study group the participant was allocated to. Clinical staff working within the hospital were also masked to participants' randomisation status. There were three primary outcomes: the proportion of paticipants who re-presented to hospital with self-harm during the 6-month follow-up period; the number of times a participant re-presented to hospital with self-harm during the 6-month follow-up period; and cost-effectiveness of the VHS as measured by estimated incremental cost per self-harm event averted. Primary outcomes were analysed in all randomised patients. Follow-up data collection was extracted from the Information Services Division of the NHS and from patient medical records. The trial is registered with International Standard Randomised Controlled Trial Number Registry, number ISRCTN99488269.

**Findings:**

Between May 9, 2012, and Feb 24, 2014, we assessed 1308 people for eligibility. Of these, 259 patients were randomly assigned to the intervention group and 259 to the control group. We obtained complete follow-up data on 512 (99%) of 518 patients (five participants were lost to follow-up in the intervention group and one in the control group). 11 patients assigned to the intervention group did not complete the VHS in hospital. Overall, the intervention did not affect the number of people who re-presented with self-harm (67 [26%] of 254 patients in the intervention group *vs* 71 [28%] of 258 patients in the control group, odds ratio [OR] 0·90, 95% CI 0·58–1·39, p=0·63). The intervention had no effect on the number of re-presentations per patient (mean 0·67 [SD 2·55] re-presentations for the intervention group *vs* 0·85 [2·79] for the control group, incident rate ratio [IRR] 1·65, 95% CI 0·74–3·67, p=0·21). Mean total costs per person for NHS hospital services in the VHS intervention group over the 6 months were £513 versus £561 in the control group but this difference was not significant (95% CI–£353 to £257, p=0·76). Three patients died by suicide in the 6 months following their index suicide attempt (one in the intervention group and two in the control group). There were no reported unintended effects or adverse events in either group.

**Interpretation:**

For the primary outcomes, there were no significant differences between groups. Although the VHS had no overall effect, post-hoc analyses suggest VHS might be effective in reducing the number of self-harm repetitions following a suicide attempt in people who complete the helpsheet and who have been previously admitted to hospital with self-harm. This is the first study to investigate the usefulness of the VHS to reduce self-harm among those who have attempted suicide. These subgroup findings require replication. The potential use of the VHS in those who self-harm for different motives requires further exploration.

**Funding:**

Chief Scientist Office (CZH/4/704).

## Introduction

Self-harm is an important predictor of death by suicide.^1^ Psychosocial interventions targeted at those who self-harm offer promise in terms of reducing self-harm repetition and suicide risk.^2^, ^3^ Although there is growing evidence for the effectiveness of long-term psychological therapies to reduce self-harm (usually in outpatient psychiatric care),^3^ few interventions have been developed specifically for acute settings. Apart from assertive case management,^4^ no brief psychological interventions based in emergency departments have been shown to be effective for those who present to emergency departments or acute services after a suicide attempt.^3^, ^5^, ^6^, ^7^, ^8^, ^9^ The aim of the present study, therefore, is to test the efficacy of an innovative brief low-intensity psychological intervention, the volitional help sheet (VHS), given in hospital within 24 h of a suicide attempt, to reduce future self-harm.

**Research in context**

**Evidence before this study**

We searched PubMed from Jan 1, 2015, to Dec 31, 2016 with the terms ‘suicid*’ or ‘self-harm’ and ‘psychological’ or ‘psychosocial’ and ‘intervention’ or ‘therapy’ for randomised controlled trials of psychological interventions to reduce repeat self-harm in patients who present to hospital following a suicide attempt.

Although there is growing evidence for the effectiveness of longer term, more intensive psychological therapies (eg, cognitive behaviour therapy and dialectical behaviour therapy) to reduce self-harm (usually in outpatient psychiatric care), few interventions have been developed specifically for administration in acute settings. There is evidence from one study that psychoeducation and case management might reduce repeat suicidal behaviour but there is no evidence for the effectiveness of brief psychological interventions administered in acute settings for patients admitted to hospital following a suicide attempt.

**Added value of this study**

Our study is the first randomised controlled trial of an implementation intentions-based brief, self-directed psychological intervention (a volitional helpsheet; VHS) developed to reduce repeat self-harm in patients who have been admitted to hospital via the emergency department following a suicide attempt. Although the intention-to-treat analyses were not significant, our post-hoc analyses suggest that a VHS might be effective in reducing the number of self-harm repetitions following a suicide attempt in people who complete it and who have been previously admitted to hospital with self-harm. However, for those with no history of self-harm hospital admission, the VHS might increase self-harm (ie, do harm), albeit the effect was not statistically significant.

**Implications of all the available evidence**

Given that the intervention is brief, it is not surprising that the effect sizes were small. However, since it is difficult to modify self-harm behaviours, the post-hoc findings for those with a past history of self-harm are important and offer promise. Since the subgroup analyses for history of past self-harm hospital admissions in those who completed the VHS following randomisation were unplanned and retrospective they require replication.

The VHS intervention is unique because it draws on theories of health behaviour change and clinical models of self-harm and suicide. The intervention uses: implementation intentions^10^ (so-called if-then plans) to promote the reduction of self-harm; the integrated motivational–volitional (IMV) model of suicidal behaviour;^11^, ^12^ research into the motives underpinning self-harm^13^, ^14^, ^15^ to identify crucial situations in which self-harm is more likely to occur;^16^, ^17^, ^18^ and processes of change derived from Prochaska and DiClemente's^19^ transtheoretical model to identify more adaptive alternative solutions to self-harm.

Implementation intentions are if–then plans that can be used to promote behaviour change by encouraging people to solve problems by explicitly linking in their memory a critical situation (ifs) with an appropriate response (thens). Laboratory research shows that when ifs are made salient, thens come automatically to mind.^10^ In the present context, an if situation could be “if I want to get relief from a terrible state of mind” and the then response would be an alternative to self-harm (eg, “…then I will think about the effect of my self-harming on the people around me”) that should make the participant more likely to choose a solution other than self-harm. According to the IMV model, implementation intentions are volitional moderators that can reduce (or increase) the likelihood of people acting on their self-injurious thoughts.^12^, ^20^

In this study, participants were encouraged to form implementation intentions by means of a VHS^21^, ^22^ that consisted of a table with two columns. One column lists theoretically derived critical situations and the other listed alternative responses to self-harm (appendix).

The VHS has already been used with some success to change other health behaviours, such as smoking and physical activity.^21^, ^22^ Additionally, in an exploratory study in Malaysia,^23^ we found that the VHS might be useful in reducing self-reported suicidal ideation and behaviour in patients. Although promising, those findings were limited by high levels of attrition and self-reported outcomes.

In this study, we aimed to investigate whether the administration of a VHS was associated with a reduction in re-presentation to hospital with self-harm (in terms of number of patients and number of re-presentations per patient) in the 6 months following an index suicide attempt. Given that repeat self-harm is most likely to occur within months of an index episode,^24^ a 6 month follow-up is optimal to capture the majority of incidences of repetition. We were also interested to identify whether the VHS affected the time to re-presentation and the extent to which it was cost effective. Since past self-harm is such a strong predictor of future self-harm and the efficacy of brief psychosocial interventions might vary as a function of self-harm history,^1^, ^25^ we also explored the extent to which past admission to hospital for self-harm moderated the effect of the VHS intervention on future self-harm.

## Methods

### Study design and participants

We did a prospective, single site, randomised controlled trial in a single hospital in Edinburgh, UK. Ethics approval was obtained from the South of Scotland Research Ethics Committee. All participants signed a written consent form and had been admitted to the Acute Medical Unit 6 (AMU6; formerly known as the Combined Assessment Area Base 6) of the Royal Infirmary of Edinburgh via the emergency department after a suicide attempt. The AMU6 is a specialist unit for patients who have been admitted overnight to hospital presenting with self-harm (ICD codes X60–X84, intentional self-harm). The trial protocol is available online.

Patients were eligible to participate in the trial if they were admitted to AMU6, had presented with a self-harm episode in which there was evidence of suicidal intent (ie, a suicide attempt), were aged 16 years or over, and had self-reported to have past history of self-harm (ie, at least one previous self-reported episode of self-harm). Patients were excluded if they were medically unfit for interview, were not competent in English, were participating in other research studies within the hospital, or had presented at the emergency department with self-harm but were subsequently discharged without hospital admission.

### Randomisation and masking

Patients were randomly assigned (1:1) to receive either the VHS intervention plus usual treatment or the control procedure (treatment as usual). Web-based randomisation was done by the Edinburgh Clinical Trials Unit by use of minimisation with a random element to ensure that the two trial groups were not significantly different on two key variables, sex and self-reported past self-harm history (1–2 previous episodes *vs* 3 or more episodes). The Information Services Division of the National Health Service (NHS-ISD) staff and those extracting data from medical notes were masked to which study group the participant was allocated to. Clinical staff working within the hospital were also masked to participants' randomisation status. The clinical staff always saw patients before the researcher, who was not masked and who delivered the intervention; therefore, it is highly unlikely that clinicians would have observed what materials the patient was completing. Most patients completed the VHS at their bedside, all with the curtain pulled across for privacy. The remainder completed the VHS in a private side room.

### Procedures

Demographic information including age, sex, marital status, employment status, and self-reported self-harm history (and associated suicidal intent) were obtained from the participants at baseline. The C-SSRS^26^ was used to assess severity and intensity of recent suicidal ideation, recent preparatory acts, and aborted and interrupted suicide attempts in the past month. The C-SSRS has been previously shown to be reliable and valid.^26^, ^27^

At baseline, all participants received treatment as usual in the first instance, which included a psychosocial assessment that was done by the Liaison Psychiatry service. After medical recovery, care depended on the results of the psychosocial assessment but could include: transfer to inpatient psychiatric care, intensive follow-up by a home treatment team, community psychiatry follow-up, specialist mental health service follow-up, third sector referral (ie, voluntary sector), or primary care follow-up. A member of the Liaison Psychiatry service made potential participants aware of the study (at the end of their assessment) and asked whether they would be interested in learning more about it, without any obligation to take part. If they agreed, a graduate psychologist researcher approached the potential participant, informing them that he or she was independent of the clinical team and that the study was investigating ways to help people to not self-harm. If the potential participant was still interested in taking part, the researcher read through the information sheet and consent form with them and answered any questions. The information sheet made it clear that they would be randomly assigned to receive either the VHS or nothing after consent. If consent was given, the participant was enrolled into the study. The same researcher recruited all of the participants to the study and adhered to a written protocol (available from the first author). Next, sociodemographic information including age, sex, marital status, employment status, and self-reported self-harm history were obtained from participants, and the Columbia–Suicide Severity Rating scale (C–SSRS)^26^ was also completed. Past self-reported self-harm history and suicidal intent of current self-harm episode were confirmed from the answers given in the C–SSRS and from the sociodemographic information. Clinicians also asked participants about suicidal intent. Participants were then randomly assigned into the intervention or control groups and informed of allocation status.

In addition to treatment as usual, participants in the intervention group received a VHS. Delivery of the intervention was standardised. Before it was administered, participants were shown a sample VHS, the researcher explained how it should be completed, and any questions were answered. The VHS began with instructions including a brief statement encouraging them to plan to stop self-harming and asked them to read through a list of common situations in which people are tempted to self-harm and a list of potential solutions (appendix). Participants were asked to draw a line linking any situation that applied to them, one solution at a time and to make as many situation-solution links as they would like. Each VHS was in duplicate form (top and carbon copy) and participants were asked to take the top copy of the VHS home with them after completion (and encouraged to refer to it at home). They were told that they might find it helpful to look at it in the future when they were feeling down and tempted to self-harm. They were reminded that they would be sent the VHS in a few months' time for follow-up. The carbon copies were used to identify whether the VHS was completed or not.

2 months after baseline, all patients in the intervention group were posted out a single booster VHS, to amplify the effect of the baseline VHS. Initially, we had planned to send out a copy of the original sheet with a summary of the solutions that participants had previously highlighted but since participants often linked numerous situations–solutions on the VHS we felt that this was confusing and difficult to summarise succinctly. As a result, we simply sent out a new blank VHS for participants to refer to, or to complete again. This was a change to the original study protocol.

We did not contact the patients again at 6 months' follow-up. The data about subsequent re-presentation with self-harm were obtained from medical records and data linkage (see Outcomes).

### Outcomes

We had three primary outcomes: the number of participants who re-presented with self-harm during the 6 month follow-up period; the number of times a participant re-presented at hospital with any self-harm during the 6 month follow-up period; and the estimated incremental cost per self-harm event averted (appendix).

The self-harm outcomes were recorded as follows: total number of self-harm re-presentations to hospital (including emergency department re-presentations that did not require inpatient treatment or overnight admissions), the number of re-presentations that were only in emergency departments, and the number of overnight self-harm admissions. If an individual presented to the emergency department but was subsequently admitted to the hospital, this was recorded only once as an overnight admission.

Our secondary outcome measure was the time to next self-harm re-presentation (in days) following randomisation.

The NHS ISD maintains a national database of hospital records and mortality data. The outcomes were extracted by NHS ISD and from patient medical records by research staff. This nationally linked database allowed us to identify whether a patient was re-admitted to hospital (ie, at least one overnight stay or admission) anywhere in Scotland with self-harm at any time since their index episode. As NHS ISD is not yet able to routinely and reliably link emergency department admissions, we had to use the medical notes for all participants (using the TRAKcare system, which covers NHS Lothian) to identify whether any participant presented to the emergency department (and was subsequently discharged) with self-harm within 6 months of their index episode. Although it is difficult to estimate the number, most baseline patients who were treated for self-harm during the study will have presented to NHS Lothian. The suicidal intent associated with self-harm was not recorded by the NHS ISD record linkage and was not planned to be included in these analyses.

### Statistical analysis

We estimated that the 6 month self-harm re-presentation rate in the control group would be approximately 20%.^28^, ^29^ Consequently, a sample size of n=259 in each group was sufficient to allow us to detect a group difference of 8% (at p<0·05 level with 80% power, one tailed) in the proportion of participants who re-presented with self-harm within 6 months (ie, 12% *vs* 20% repetition). Such a difference represents a clinically significant reduction in self-harm rates between the groups.^25^, ^30^

We report intention-to-treat analyses (ie, everyone who was randomised) and post-hoc analyses of everyone who completed the VHS in hospital following randomisation for all primary outcomes. NHS ISD successfully linked 512 (99%) of 518 randomised participants from both groups (five in the intervention group *vs* one in the control group were not linked). We were able to identify emergency department re-presentations (via medical notes) for all patients. Logistic regression analyses and negative binomial models were used to investigate the association between the number of participants re-presenting to hospital with self-harm or frequency of re-presentations with exposure to the intervention (appendix). We used logistic analysis and negative binomial models to probe whether past history of hospital admission moderated treatment effects.^31^, ^32^ After randomisation, 11 (4%) of 259 participants in the intervention group did not complete the VHS intervention in hospital, therefore we did post-hoc analyses on the 248 participants who received the intervention in hospital. The 11 participants did not complete the VHS for various reasons, usually because they were too tired or upset to complete it. An additional two participants did not receive the 2-month booster intervention but were included in the analyses because they completed the VHS in hospital. We did Cox proportional survival analyses to investigate the extent to which the intervention affected the time to re-presentation. Since past self-harm is a powerful predictor of future self-harm and can influence the efficacy of the intervention,^1^, ^25^, ^33^ in our models we also included admission to hospital for self-harm in the 10 years preceding baseline (past self-harm hospitalisation) as a predictor and moderator of the intervention. The analysis of participants who completed the VHS in hospital after randomisation and the past self-harm hospitalisation moderation analyses are post-hoc subgroup analyses.

It was not possible to identify whether the six unlinked participants had been admitted to hospital with self-harm in the previous 10 years. Of the total sample of 512 who were linked, 183 (35%) had not been admitted to hospital following self-harm in the previous 10 years. Of the 329 participants who had been admitted to hospital with self-harm, 123 (37%) had only done so once, with the remaining 206 ranging from two to 72 times. Since the distribution was highly skewed, we dichotomised this variable into those who had never self-harmed and those who had self-harmed at least once.

In the intention-to-treat analysis for the six participants who NHS ISD was unable to link, we used the most conservative strategy of assuming that they had been admitted once overnight with self-harm during the follow-up period. For the negative binomial models, we imputed the missing 10 year dichotomous self-harm history variable using logit models (appendix). All models were based on a 20 imputation run and estimated in Stata version 13 using multiple imputation chained equations.^34^ We report incident rate ratio (IRR) and unstandardised coefficient (B) for both the available listwise data and imputed models to include the six missing participants.

For the economic analysis, we calculated the incremental cost per self-harm event averted for the intervention compared with the control group for the intention-to-treat analysis and the post-hoc analysis of those who completed the VHS in hospital after randomisation for both listwise and imputed data. We also did sub-group analyses for individuals who had or had not been admitted to hospital for self-harm in the previous 10 years for the intention-to-treat and post-hoc subgroup samples. We did the analysis from a health-service perspective attaching relevant unit costs to all self-harm events identified via NHS ISD and medical records. These costs included treatment within emergency departments and costs associated with admission to hospital after injury. We also present decision-making willingness to pay bootstrapping analyses. Full details are described in the appendix. The trial is registered with ISRCTN, number 99488269.

### Role of the funding source

The funder had no role in study design, data collection, data analysis, data interpretation, or writing of the report. The corresponding author had full access to all the data in the study and had final responsibility for the decision to submit for publication.

## Results

Of 1308 people assessed for eligibility, we recruited 518 participants between May 9, 2012, and Feb 24, 2014. Of these, 259 were randomly assigned to the intervention group and 259 to the control group (figure). Follow-up data collection covered the time period of November, 2012, until August, 2014. Table 1 shows the characteristics of the sample at baseline. Three patients died by suicide in the 6 months following their index suicide attempt (one in the intervention group and two in the control group). There were no unintended effects or adverse events in either group.

**Figure:**
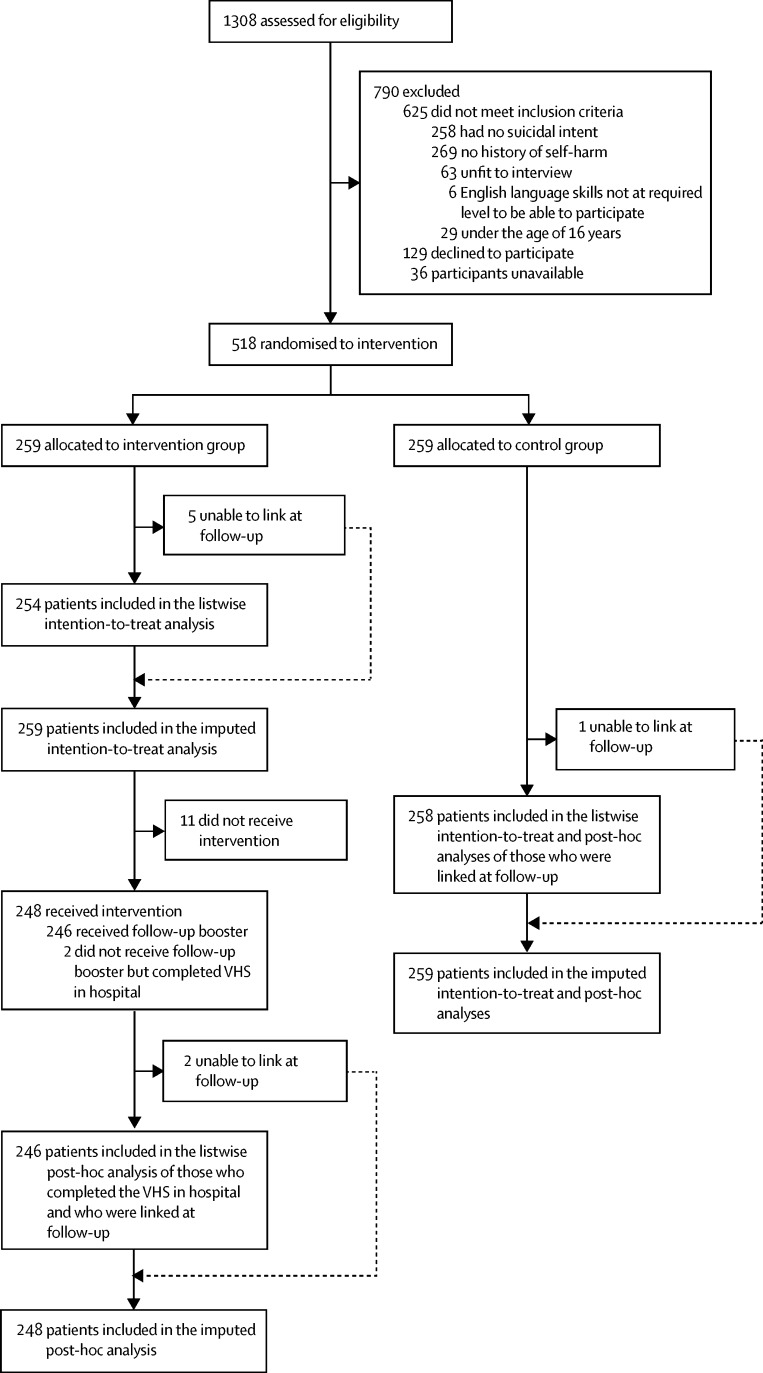
Trial profile

**Table 1:** Baseline characteristics

	**Intervention group (n=259)**	**Control group (n=259)**
Mean age, years	36·50 (14·59)	36·07 (12·77)
Male	99 (38%)	95 (37%)
Female	160 (62%)	164 (63%)
Married/de-facto	58 (23%)	57 (22%)
Unemployed or receiving social security benefits	165 (65%)	180 (70%)
Self-poisoning	253 (98%)	251 (97%)
Previous self-harm overnight admissions in the past 10 years	161 (63%)	168 (65%)
Mean severity of suicidal ideation, Likert-type scale	3·55 (1·55)	3·49 (1·69)
Mean intensity of suicidal ideation	2·93 (0·81)	3·01 (0·77)
Mean lifetime previous suicide attempts	1·65 (0·88)	1·54 (0·93)
Mean interrupted attempts in the past month	0·37 (1·00)	0·34 (1·02)
Mean aborted attempts in the past month	0·77 (1·47)	0·52 (1·10)
Mean preparatory acts in the past month	1·63 (0·50)	1·72 (0·49)

Data are given as mean (SD) or n (%). Lifetime previous suicide attempts was recorded as follows: 0=no previous attempts, 1=1–2 previous attempts, 2=3–4 previous attempts, 3=5 or more previous attempts.

The proportion of participants who re-presented with self-harm are summarised in table 2. The intervention did not affect the number of people who re-presented with self-harm after 6 months' follow-up (table 3). There was also no evidence for moderation (interaction of group by self-harm hospital admission in the past 10 years).

**Table 2:** Re-presentation for self-harm in the 6 months following the index presentation

		**Intervention group**		**Control group**	
		Listwise (n=254)	Imputed (n=259)	Listwise (n=258)	Imputed (n=259)
Re-presentation for self-harm					
	Admitted overnight to general hospital*	49 (19%)	54 (21%)	54 (21%)	55 (21%)
	Presented at emergency department but discharged†	34 (13%)	34 (13%)	38 (15%)	38 (15%)
	Overall, any self-harm (overnight hospitalisation or emergency department presentation)	67 (26%)	72 (28%)	71 (28%)	72 (28%)
Number of self-harm re-presentations					
	Admitted overnight to general hospital*	0·35 (1·26)	0·37 (1·26)	0·37 (1·13)	0·37 (1·13)
	Presented at emergency department but discharged (ie, not admitted to hospital)†	0·32 (1·43)	0·32 (1·43)	0·49 (1·95)	0·49 (1·95)
	Total (overnight hospitalisation or emergency department presentation)	0·67 (2·55)	0·68 (2·53)	0·85 (2·79)	0·85 (2·78)
Time to re-presentation		61·54 (50·05)	61·71 (48·26)	66·28 (54·49)	66·25 (54·11)

Data are given as n (%) or mean (SD). Listwise refers to participants for whom follow-up data was collected.

* Imputed numbers include n=5 (intervention) and n=1 (control) unlinked participants assumed to have been admitted once to hospital overnight with self-harm.

† Listwise and imputed are the same as no emergency department data were missing.

**Table 3:** Logistical regression analyses investigating association between group assignment (intervention *vs* control), past admission to hospital for self-harm, and re-presentation with self-harm

	**Intention-to-treat analysis**				**Subgroup analysis**			
	Listwise (n=512)		Imputed (n=518)		Listwise (n=504)		Imputed (n=507)	
	OR (95% CI)	p value	OR (95% CI)	p value	OR (95% CI)	p value	OR (95% CI)	p value
**Overall re-presentations**								
Group (intervention *vs* control)	0·90 (0·58–1·39)	0·63	0·86 (0·56–1·32)	0·50	0·96 (0·62–1·48)	0·84	0·94 (0·61–1·46)	0·79
Hospital admission for self-harm in past 10 years	1·69 (1·10–2·61)	0·018	1·80 (1·17–2·77)	0·0070	1·57 (1·01–2·43)	0·044	1·62 (1·05–2·51)	0·030
Group × self-harm in past 10 years	2·18 (0·92–5·20)	0·077	2·00 (0·84–4·73)	0·12	2·54 (1·06–6·11)	0·037	2·46 (1·03–5·89)	0·043
**Emergency department**								
Group (intervention *vs* control)	0·93 (0·54–1·63)	0·81	0·95 (0·54–1·65)	0·84	1·02 (0·58–1·80)	0·95	1·02 (0·58–1·80)	0·94
Hospital admission for self-harm in past 10 years	1·43 (0·82–2·50)	0·20	1·40 (0·81–2·45)	0·23	1·30 (0·74–2·28)	0·37	1·28 (0·73–2·26)	0·39
Group × self-harm in past 10 years	2·41 (0·79–7·34)	0·12	2·48 (0·82–7·54)	0·11	2·96 (0·95–9·16)	0·061	2·98 (0·96–9·23)	0·059
**Overnight admission**								
Group (intervention *vs* control)	1·00 (0·62–1·62)	0·99	0·94 (0·58–1·52)	0·81	1·07 (0·65–1·74)	0·80	1·04 (0·64–1·70)	0·87
Hospital admission for self-harm in past 10 years	1·74 (1·07–2·82)	0·026	1·88 (1·16–3·04)	0·010	1·61 (0·98–2·62)	0·058	1·68 (1·03–2·73)	0·037
Group × self-harm in past 10 years	1·56 (0·59–4·10)	0·37	1·39 (0·53–3·63)	0·50	1·82 (0·68–4·85)	0·23	1·74 (0·66–4·62)	0·26

Subgroup analyses are of participants who completed the VHS in hospital after randomisation.

The number of self-harm re-presentations per patient did not differ between groups, and the analysis produced the same results for both the listwise and imputed models (Table 2, Table 4). The 10-year history of previous self-harm hospital admission significantly predicts increases in the incidence of subsequent self-harm. The group by 10-year history of previous self-harm interaction was significant for emergency department re-presentations (table 4).

**Table 4:** Negative binomial models to predict repeat self-harm as function of group and past admission to hospital for self-harm

	**Intention-to-treat analysis**						**Sub-group analysis**					
	Listwise (n=512)			Imputed (n=518)			Listwise (n=504)			Imputed (n=507)		
	IRR (95% CI)	B (95% CI)	p value	IRR (95% CI)	B (95% CI)	p value	IRR (95% CI)	B (95% CI)	p value	IRR (95% CI)	B (95% CI)	p value
**Emergency department**												
Intervention (0=control, 1=treatment)	2·11 (0·65 to 6·77)	0·75 (−0·42 to 1·91)	0·21	2·05 (0·65 to 6·62)	0·72 (−0·43 to 1·89)	0·22	2·13 (0·68 to 6·67)	0·76 (−0·38 to 1·89)	0·19	2·11 (0·68 to 6·62)	0·75 (−0·38 to 1·89)	0·19
Self-harm in the past 10 years (0=never, 1=at least once)	5·55 (1·99 to 15·42)	1·71 (0·69 to 2·73)	0·0010	5·53 (1·99 to 15·33)	1·71 (0·69 to 2·73)	0·0010	5·55 (2·00 to 15·06)	1·71 (0·71 to 2·71)	0·0010	5·53 (2·00 to 14·03	1·71 (0·71 to 2·71)	0·001
Intervention × self-harm in the past 10 years interaction	0·25 (0·06 to 0·99)	−1·40 (−2·78 to −0·09)	0·049	0·25 (0·06 to 0·99)	−1·39 (−2·78 to −0·004)	0·049	0·15 (0·04 to 0·59)	−1·91 (−3·29 to −0·53)	0·0070	0·15 (0·004 to 0·59)	−1·91 (−3·29 to −0·53)	0·007
Intercept	0·12 (0·05 to 0·29)	−2·1 (−2·98 to −1·21)	<0·0001	0·12 (0·05 to 0·29)	−2·10 (−2·99 to −1·22)	<0·0001	0·12 (0·05 to 0·29)	−2·10 (−2·97 to −1·23)	<0·0001	0·12 (0·05 to 0·29)	−2·10 (−2·97 to −1·24)	<0·0001
Ln (α)	2·31 (1·97 to 2·64)	2·31 (1·97 to 2·64)	<0·0001	2·32 (1·98 to 2·66)	2·32 (1·98 to 2·66)	<0·0001	2·23 (1·87 to 2·59)	2·23 (1·87 to 2·59)	<0·0001	2·24 (1·88 to 2·60)	2·24 (1·88 to 2·60)	<0·0001
**Overnight hospitalisation**												
Intervention (0=control, 1=treatment)	1·36 (0·58 to 3·19)	0·31 (−0·54 to 1·16)	0·47	1·42 (0·62 to 3·22)	0·35 (−0·47 to 1·17)	0·40	1·38 (0·60 to 3·17)	0·32 (−0·50 to 1·15)	0·45	1·38 (0·61 to 3·13)	0·32 (−0·49 to 1·14)	0·44
Self-harm in the past 10 years (0=never, 1=at least once)	2·45 (1·18 to 5·11)	0·90 (0·16 to 1·63)	0 016	2·43 (1·20 to 4·95)	0·89 (0·18 to 1·60)	0·014	2·45 (1·20 to 5·03)	0·89 (0·18 to 1·61)	0·014	2·41 (1·20 to 4·85)	0·88 (0·18 to 1·58)	0·014
Intervention × self-harm in the past 10 years interaction	0·64 (0·23 to 1·77)	−0·43 (−1·44 to 0·57)	0 40	0·63 (0·24 to 1·68)	−0·45 (−1·43 to 0·52)	0·36	0·46 (0·17 to 1·26)	−0·76 (−1·76 to −0·23)	0·13	0·47 (0·18 to 1·26)	−0·75 (−1·73 to 0·23)	0·13
Intercept	0·19 (0·10 to 0·35)	−1·66 (−2·29 to −1·03)	<0·0001	0·19 (0·10 to 0·36)	−1·65 (−2·27 to −1·03)	<0 0001	0·19 (0·10 to 0·35)	−1·66 (−2·28 to −1·05)	<0·0001	0·19 (0·10 to 0·35)	−1·64 (−2·25 to −1·04)	<0·0001
Ln (α)	1·38 (1·03 to 1·74)	1·38 (1·03 to 1·74)	<0·0001	1·29 (0·94 to 1·64)	1·29 (0·94 to 1·64)	<0·0001	1·28 (0·89 to 1·67)	1·28 (0·89 to 1·67)	<0·0001	1·23 (0·84 to 1·62)	1·23 (0·84 to 1·62)	<0·0001
**Total self-harm re-presentations**												
Intervention (0=control, 1=treatment)	1·65 (0·74 to 3·67)	0·51 (−0·29 to 1·30)	0 21	1·68 (0·77 to 3·63)	0·52 (−0·26 to 1·29)	0·19	1·67 (0·77 to 3·64)	0·52 (−0·26 to 1·29)	0·19	1·66 (0·78 to 3·60)	0·51 (−0·25 to 1·28)	0·19
Self-harm in the past 10 years (0=never, 1=at least once)	3·67 (1·83 to 7·35)	1·30 (0·60 to 1·99)	0·0001	3·63 (1·91 to 7·10)	1·29 (0·65 to 1·97)	<0·0001	3·67 (1·86 to 7·22)	1·30 (0·62 to 1·98)	<0·0001	3·60 (1·86 to 7·03)	1·28 (0·62 to 1·95)	<0 0001
Intervention × self-harm in the past 10 years interaction	0·40 (0·15 to 1·05)	−0·91 (−1·86 to 0·48)	0·063	0·40 (0·16 to 1·02)	−0·91 (−1·85 to 0·02)	0·054	0·27 (0·10 to 0·68)	−1·32 (−2·26 to −0·37)	0·0060	0·27 (0·10 to 0·68)	−1·31 (−2·24 to −0·38)	0·006
Intercept	0·31 (0·17 to 0·56)	−1·17 (−1·76 to −0·57)	<0·0001	0·32 (0·17 to 0·56)	−1·14 (−1·74 to −0·58)	<0·0001	0·31 (0·17 to 0·55)	−1·17 (−1·74 to −0·59)	<0 0001	0·31 (0·18 to 0·55)	−1·16 (−1·72 to −0·59)	<0·0001
Ln (α)	1·60 (1·35 to 1·85)	1·60 (1·35 to 1·85)	<0·0001	1·54 (1·29 to 1·79)	1·54 (1·29 to 1·79)	<0·0001	1·67 (0·77 to 3·64)	0·52 (−0·26 to 1·29)	0·19	1·66 (0·78 to 3·60)	0·51 (−0·25 to 1·28)	0·19

B=unstandardised coefficient. IRR=incident rate ratio. Subgroup analyses are of participants who completed the VHS in hospital after randomisation.

Based on the listwise data, in the control group those who had been admitted to hospital previously (for self-harm) self-harmed more frequently than those who had not been previously admitted to hospital (IR=5·5, 95% CI 1·99–15·4, p=0·0010). None of the other comparisons were significant. Thus, in the intervention group the incidence of self-harm for those previously admitted to hospital for self-harm is no longer different from those with no previous history.

Cox proportional survival analyses revealed no significant differences in time (in days) to next self-harm episode between the groups for both listwise (hazard ratio [HR] 0·81, 95% CI 0·55–1·20, p=0·30) and imputed analyses (HR 0·80, 0·55–1·18, p=0·26) and this difference was not moderated by past history of admission to hospital for self-harm in either listwise (HR 1·45, 0·67–3·13, p=0·35) or imputed analyses (HR 1·44, 0·67–3·1, p=0·35). The same null findings were found when analyses were limited to the sub-group of those who completed the VHS following randomization (not reported).

Mean total costs per person for NHS hospital services in the VHS intervention group over the 6-month period were £513 compared with £561 in the control group but this difference was not significant (95% CI –£353 to £257, p=0·76; appendix). The results of logistical regression comparing the means of overall costs per person in the intention-to-treat, those participants who completed the VHS, and 10-year history of admission to hospital for self-harm sub-group analyses also found no significant differences in mean costs between groups. Mean costs for the past 10-year self-harm admitted to hospital sub-group analysis in participants who completed the VHS was £428 in the intervention group and £717 in the control group (difference in means £289, 95% CI=–672 to 93, p=0·14, appendix).

With the exception of those who had not been previously admitted to hospital, the results of all bootstrapping analyses indicate that the intervention is dominant compared with treatment as usual alone, with lower mean costs for the intervention that are very similar to those reported in the regression analysis (appendix). The bootstrapped analysis indicates that there is always a more than 50% chance of the VHS being considered cost effective compared with treatment as usual even when the willingness to pay is zero or very low (appendix). The economic case appears most promising for the bootstrapped analysis of the post-hoc analysis of those individuals who completed the VHS in hospital and who have a history of self-harm hospital admission in the past 10 years. For this group, there is more than a 90% likelihood that when administering the intervention, the cost per additional self-harm case averted will be less than potential willingness to pay (appendix).

Consistent with the intention-to-treat analyses, there was no main effect of the intervention on the proportion of participants who re-presented with self-harm when the post–hoc analyses were limited to those who completed the VHS (table 3). The group by self-harm admission to hospital in the past 10 years interaction was significant for overall re-presentations when analysed listwise (OR 2·54, 95% CI 1·06–6·11, p=0·037) and with imputed data (2·46, 1·03–5·89, p=0·043). Decomposition of the interaction shows that in the absence of treatment, those with a previous history of self-harm were significantly more likely to re-present with self-harm (difference in re-presentation 16·6%, 95% CI 5·17–28·03, p=0·0050). Additionally, among participants with a history of self-harm, there was no significant effect of treatment leading to a decreased proportion of participant re-presentation (difference in re-presentation 8·6%, 95% CI −1·34 to 18·54, p=0·087). Nonetheless, in terms of numbers needed to treat, one in every 12 patients treated should show benefit. However, for those with no history of admission to hospital for self-harm, although there was no significant effect of treatment (difference in re-presentation −8·3%, 95% CI −3·51 to 20·11, p=0·17), one in 13 might be harmed (ie, might re-present with self-harm).

The listwise and imputed models for participants who completed the VHS gave the same results for the number of re-presentations to hospital (table 4). The 10-year history of previous self-harm is significantly associated with an increase in the incidences of subsequent self-harm across all of the outcomes and it significantly moderates the effect of the intervention for emergency department re-presentations and the total outcome.

The listwise analyses show that for those who have been hospitalised for self-harm in the past 10 years, their levels of subsequent self-harm are significantly lower by 69% when exposed to the intervention (IRR=0·31, 95% CI 0·14–0·71, p=0·0050). Also, patients in the control group who had been admitted to hospital for self-harm in the previous 10 years were significantly more likely to present to the emergency department with subsequent self-harm (IRR 5·55 1·99–15·44, p=0·0010). All other comparisons were non-significant. The same pattern of findings is evident for the total outcome; for those patients who had self-harmed previously in the last 10 years, their rate of subsequent self-harm was 55% lower when exposed to the intervention (IRR 0·45, 0·26–0·77, p=0·0040). Patients in the control group who had been admitted to hospital for self-harm in the previous 10 years were significantly more likely to engage in subsequent self-harm (IRR 3·67, 1·83–7·35, p=0·0010).

## Discussion

This randomised controlled trial investigated the efficacy of a VHS to reduce self-harm in patients who had presented to hospital following a suicide attempt. The intervention had no overall effect but the efficacy of the intervention might be associated with 10-year history of admission to hospital for self-harm.

Taken as a whole, the findings fit with precision medicine approaches that highlight the vital importance of tailoring treatment to the individual patient, in this case suggesting that different approaches are required for those with and without a history of self-harm hospitalisation. The findings for the number of self-harm episodes are promising for participants who completed the VHS intervention in hospital. Although there was no main effect of the intervention on the number of self-harm episodes overall, there was evidence that the VHS might be of use in reducing the number of self-harm episodes in participants who had been admitted to hospital with self-harm in the past 10 years. In the post-hoc analyses of participants who completed the intervention in hospital, exposure to the VHS was associated with a 69% reduction in the number of emergency department self-harm re-presentations and a 55% reduction in the total number of hospital re-presentations. The VHS intervention did not have any effect on the time to re-presentation to hospital with self-harm. The mean total costs per person for NHS hospital services in the VHS intervention group over the 6 months were not statistically different from those in the control group. For the intention-to-treat analysis, there is a more than 50% chance of the intervention being considered cost effective for all potential levels of willingness to pay for averting a self-harm case. The economic case appears most promising if it is targeted at those with a history of being admitted to hospital for self-harm for which there is more than a 90% chance of the intervention being cost effective.

The subgroup findings are consistent with a recent Cochrane review,^3^ which suggests that for more intensive interventions, for which trials have been shown to be effective, they tend to be among those with a history of self-harm. It would also be helpful to identify in a subsequent trial what role the booster VHS played, what participants thought about the VHS and to what extent they completed it again. Additionally, although the treatment effect was not statistically significant in participants with no previous self-harm admission to hospital, it is crucial that we establish whether a brief adjunct intervention, like a VHS, might be associated with harm (ie, increased re-presentation with self-harm) in this group—and why. Within the current study design, we were unable to systematically investigate the extent to which the VHS is effective for those who self-harm with high frequency.

Although the study had many strengths, its potential limitations need to be considered. First, the study was powered to detect a clinically meaningful reduction in the proportion of people who would self-harm, but it was not powered on the repetition rate for sub-group analyses or to detect differences in costs. Additionally, the past self-harm admission to hospital moderator analyses were unplanned, data-driven, and influenced by the extant literature;^25^ therefore these retrospective sub-group analyses require replication since they are beyond our planned intention-to-treat analyses. Second, since we did not assess self-harm in the community at follow-up, we cannot generalise our findings beyond hospital-treated self-harm. Although it is unlikely to have affected the findings, we were only able to identify whether participants presented to the emergency department in one health board area during the follow-up; so there is the possibility that we missed a small number of emergency department re-presentations elsewhere in Scotland or beyond. Although beyond the scope of the present research, future studies should also seek to measure effects on the use of primary care and community mental health services related to self-harm during the follow up period.

Building on the present findings, we should identify the extent to which the VHS is generalisable to other self-harm subgroups including those who present with non-suicidal self-harm and those who are discharged from hospital following treatment at the emergency department. Although this study was done at a single site, in a large general hospital with an acute medical unit, there is no reason to question the generalisability of our findings to other hospital-treated suicide attempt populations.

In conclusion, although the VHS had no overall effect, it might be effective in reducing self-harm in people who have attempted suicide and have a history of self-harm hospital admission. Since the sub-group analyses for past self-harm hospitalisation and VHS completion were unplanned and retrospective they require replication, especially given the potential negative effect in those with no self-harm hospitalisation history.
