# Supplementary material for: A brief psychological intervention to reduce repetition of self-harm in patients admitted to hospital following a suicide attempt: a randomised controlled trial
Source: Lancet Psychiatry. 2017 Jun;4(6):451–60. doi: 10.1016/S2215-0366(17)30129-3 (PMC5447136; doi:10.1016/S2215-0366(17)30129-3)
Supplement: Supplementary appendix [file mmc1.pdf]

THE LANCET
Psychiatry

Supplementary appendix

This appendix formed part of the original submission and has been peer reviewed. We post it as supplied by the authors.

Supplement to: O'Connor RC, Ferguson E, Scott F, et al. A brief psychological intervention to reduce repetition of self-harm in patients admitted to hospital following a suicide attempt: a randomised controlled trial. *Lancet Psychiatry* 2017; published online April 20. http://dx.doi.org/10.1016/S2215-0366(17)30129-3.

Appendix

1. The Volitional Help Sheet

Help Sheet

We want you to plan to avoid self-harming. Research shows that if people can spot situations in which they will be tempted to self-harm and then link them with a way to overcome those situations, they are much more likely to be successful in avoiding self-harming.

On the left hand side of the page below is a list of common situations in which people feel tempted to self-harm; on the right hand side of the page is a list of possible solutions.

For each situation that applies to you personally (left hand side), ✎ **please draw a line linking it to a solution** (right hand side) that you think might work for you. Please draw a line linking one situation to one solution at a time, **but make as many (or as few) situation-solution links as you like. There is a blank situation box and a blank solution box at the bottom for you to include anything else which might also apply to you.**

SITUATIONS		Please draw lines to the boxes that are relevant		SOLUTIONS
If I am tempted to self-harm when I want to get relief from a terrible state of mind ...	☐		☐	... then I will do something else instead of self-harming
If I am tempted to self-harm when I want to punish myself ...	☐		☐	... then I will tell myself that I can stop self-harming if I want to
If I am tempted to self-harm when I want to die ...	☐		☐	... then I will recall information people have given me about the benefits of stopping self-harming
If I am tempted to self-harm when I want to show how desperate I am feeling ...	☐		☐	... then I will tell myself that Society is changing in ways that make it easier for people to stop self-harming
If I am tempted to self-harm when I want to find out whether someone really loves me ...	☐		☐	... then I will make sure I am rewarded by others if I don't self-harm
If I am tempted to self-harm when I want to get some attention ...	☐		☐	... then I will think about the impact of my self-harming on the people around me
If I am tempted to self-harm when I want to frighten someone ...	☐		☐	... then I will remember that I react emotionally to warnings about my self-harming
If I am tempted to self-harm when I want to get my own back on someone	☐		☐	... then I will remember that I get upset when I think about my self-harming
If I am tempted to self-harm when I feel defeated ...	☐		☐	... then I will put things around my home or place of work that remind me not to self-harm
If I am tempted to self-harm when I feel trapped ...	☐		☐	... then I will seek out someone who listens when I need to talk about self-harm
If I am tempted to self-harm when I feel hopeless ...	☐		☐	... then I will take medication
If I am tempted to self-harm when I _______________ _______________________________________ ...	☐		☐	... then I will _______________________________ _______________________________________

2. Additional Information on Analysis

Each of the three self-harm outcomes (ED re-presentations, overnight hospitalisations, total self-harm re-presentations) constitutes count data that are over-dispersed (Pearson's coefficient of over-dispersion = 6·0. 3·6, and 8·2 respectively), as such standard Poisson regression models are not appropriate. These over-dispersed distributions were characterised and likely caused by a high incidence of zero counts (446/518 for ED re-presentations, 409/512 for overnight hospitalisations and 374/512 for total re-presentations). To deal with the degree of over-dispersion we fitted negative binomial models. We also fitted zero-inflated negative binomial models but these were statistically not as good a fit to these data as the negative binomial fit. Conceptually, also it is unlikely that there are zero incidents of self-harm because the person is unable to self-harm.

Additional information on missing data and imputation

We imputed the missing 10 year dichotomous self-harm history variable using logit models from randomization, the outcome of interest, sex and self-reported history of self-harm (sex and self-reported history of self-harm were auxiliary variables not included in the main negative binomial models). With such a small amount of missing data 1·2% (n=6) for the ITT and 0·6% (n=3) for the PP analyses, listwise deletion should give the same results as the imputed data.

3. Health economic analysis

Aims

The aim of the economic analysis was to identify incremental cost per self-harm event averted over a six month follow-up period as a result of using the VHS intervention. The analysis was conducted from the perspective of the Scottish NHS. As both the intervention and control groups are assumed to initially receive the same treatment as usual within the Edinburgh Royal Infirmary, the analysis estimated the resource use and costs associated with subsequent use of accident and emergency services and inpatient hospital stays related to further self harm events. Admissions to hospital for self-harm were obtained from the Information Services Division of the NHS National Services Scotland (NHS ISD) national database of hospital records. The medical notes of all study participants were examined in order to identify contacts with Emergency Department (ED) services for self-harm. The nature of this study meant that it was not possible to ask individuals to complete a self-report health care utilisation questionnaire at time of hospital admission or subsequently; so community-based mental health treatment or primary care services were not included in the analysis.

Calculation of costs

A bespoke approach was used to identify costs associated with the development of the VHS and its implication. The routine costs of implementation were estimated based on consultation with clinicians at the Edinburgh Royal Infirmary. In our economic analysis we have only included the costs associated with routine implementation of the VHS, excluding any development costs.

The VHS can be administered in an acute medical unit by a nurse within 10 minutes. This time was valued using the hourly wage rates for a Band 5 nurse in Scotland (https://www.rcn.org.uk/employment-and-pay/nhs-pay-scales-2014-15). In addition there are minor postage costs and packaging costs for the follow-up booster VHS. A nurse will also require one-off training of no more than 3 hours training before making use of the VHS and these training costs have been apportioned over the study population to identify training costs per person of £0.13. Overall costs come to £4.38 per contact which we have conservatively rounded up to £5.

Costs to the health care system of dealing with self-harm events in our analysis include the costs of emergency ambulance transportation, treatment within EDs and costs associated with hospitalisation following injury (see Table A1). All unit cost data are taken from the 2014 Scottish Health Services Costs book[1] published by the Information Services Division of NHS Scotland, with the exception of the cost of psychosocial assessments considered in sensitivity analysis which are valued using the English 2014 Mental Health Cluster for Initial Assessment Tariff for common mental health problems (low severity)[2]. A proportion of all individuals who attend an ED following self-harm events will arrive by ambulance. We were not able to identify this for the study population but have assumed that this is same as for the general population in Scotland: approximately 24% [3]. The cost per incident to the Scottish Ambulance Service in their south-east operating division covering Borders and Lothian of £231.47 is used in this analysis. The average cost per ED attendance at the Edinburgh Royal Infirmary is £106.

There will be significant variation in the costs of hospitalisation related to self-harm, for instance from just a few hundred pounds for the treatment of poisoning and wounds without complications to many thousands of pounds related to major trauma and subsequent need for ongoing care related to rehabilitation. We do not have data in this analysis on the injury sustained for each subsequent hospitalisation; we have assumed that 80% of self-harm hospitalisations will be to deal with poisonings, in line with data reported in England[4]. We have used national

[1] For 2014 costs book see http://www.isdscotland.org/Health-Topics/Finance/Costs/File-Listings-2014.asp

[2] See https://www.gov.uk/government/publications/nhs-reference-costs-2013-to-2014 MHCCIA Cluster 01
[3] NHS Scotland. Understanding emergency care in NHS Scotland. Patient pathways through the emergency department. Edinburgh: ISD Scotland, January 2016.
[4] Hawton K, Haw C, Casey D et al. Self-harm in Oxford, England: epidemiological and clinical trends, 1996–2010. Soc Psychiatry Psychiatr Epidemiol (2015) 50:695–704

tariff rates in Scotland for poisoning, toxic, environmental and unspecified effects with intermediate complications for individuals who are admitted to hospital (£765). For the remaining 20% of cases we have conservatively used the average cost for any inpatient case at the Edinburgh Royal Infirmary of £2,721; in fact costs for treatment of many severe wounds are more than double this cost.

Table A1. Summary of unit costs in analysis

Description of service	Unit	Cost	Source
Intervention: VHS	Per intervention group participant	£5	Expert opinion re: time to administer VHS; Royal College of Nursing Hourly Salary Scales Scotland for Band 5 Nurse 2014; observed cost of producing VHS, plus materials postage packaging & training
Ambulance to ED	Per attendance	£231.47	Information Services Division (ISD), NHS Scotland National Statistics 2014
ED consultation only	Per attendance	£106	Information Services Division (ISD), NHS Scotland National Statistics 2014, Edinburgh Royal Infirmary Hospital Running Costs
Hospitalisation (except poisoning, toxic, environmental or other unspecified effects)	Per hospitalisation	£2721	Information Services Division (ISD), NHS Scotland National Statistics 2014, Edinburgh Royal Infirmary Hospital Running Costs
Hospitalisation for poisoning, toxic, environmental or other unspecified effects)	Per hospitalisation	£765	Information Services Division (ISD) NHS Scotland, 2013/14 Scottish Tariffs for Cross Boundary Flow Costing. Non elective inpatient cost, with intermediate complications
Psychosocial assessment	Per hospitalisation	£256	Reference Cost Collection: National Schedule of Reference Costs - Year 2013-14 - NHS trusts and NHS foundation trusts. Mental Health Cluster Initial Assessment (MHCCIA01)

All costs are reported using 2013/14 prices

Data analysis

Statistical analyses were conducted using Stata version 14. Linear regression analysis was used to compare differences in mean costs of subsequent self-harm events between the treatment and control groups. Non-parametric bootstrapping (1000 replications), resampling pairs of costs and outcomes was performed. This is a standard approach for assessing the robustness of these results.[1] It produces a distribution of possible mean costs and effects that were also visualised on a cost effectiveness plane to aid decision-making, reflecting the level of variability in the trial.[2] These distributions were then used to explore the likelihood that VHS plus treatment as usual would be preferable to treatment as usual given different levels of investment that NHS decision-makers might be willing to invest per additional self-harm case averted.[3]

Given the small number of suicides (n=3), we have not conducted any economic analyses on these data.

Cost effectiveness analysis

Table A2 provides an overview of estimated costs in both the VHS and control groups for the intention to treat analysis. Total costs to the NHS in the VHS group over the six month period are estimated to be £132,979 compared with £145,381 for the control group, with mean costs of £513 and £561. All elements of cost: use of ambulances, attendance at the ED and hospitalisations have lower mean values. The results of logistic regression in Table A3 comparing the means of overall costs for all ten analyses run, including those who completed the VHS following randomisation and past self-harm hospitalisation sub-group analyses, indicate that there are no statistically significant differences in mean costs between groups. With the exception of sub-group analyses for those participants who had no history of self-harm hospitalisation in the previous ten years, mean costs are lower in the VHS than in the control group.

Table A2: Overview of health system and productivity costs for VHS and control groups (ITT analysis)

Group membership	Minimum	Maximum	Sum	Mean	Std. Deviation

**VHS Group (n=259)**	**VHS**	5	5	1,295	5	0
	**Ambulances**	0	1,833	9,500	37	140
	**Attendance at ED**	0	2,014	8,586	33	150
	**Attendance at ED followed by hospitalisation**	0	17,671	113,598	439	1,581
	**Total NHS Costs**	5	21,523	132,979	513	1,837
**Control Group (n=259)**						
	**VHS**	0	0	0	0	0
	**Ambulances**	0	1,611	12,222	47	155
	**Attendance at ED**	0	2,120	13,250	51	207
	**Attendance at ED followed by hospitalisation**	0	16,408	119,909	463	1,424
	**Total NHS Costs**	0	19,716	145,381	561	1,696

Table A3. Differences in mean NHS costs between VHS and control groups for each analysis

**Analysis**	**Co-efficient**	**95% Confidence Interval**	**p-value**
ITT (n=518)	-47.89	(-353.10 to 257.33)	0.758
ITT – Imputed (n=518)	-28.39	(-333.75 to 276.97)	0.855
Completers (n= 507)	-147.24	(-411.25 to 116.65)	0.274
Completers – Imputed (n=507)	-141.93	(-405.96 to 122.10)	0.291
ITT – those with a self-harm history (n=335)	-132.77	(-580.56 to 315.34)	0.560
ITT – Imputed – those with a self-harm history (n=335)	-102.22	(-550.34 to 345.89)	0.654
ITT – those without a self-harm history (n=183)*	118.10	(-149.00 to 385.21)	0.384
Completers – those with a self-harm history (n=325)	-289.42	(-671.99 to 93.13)	0.138
Completers – Imputed – those with a self-harm history-history (n=325)	-280.71	(-663.43 to 102.00)	0.150
Completers – those without a self-harm history (n=182)*	122.25	(-146.19 to 390.70)	0.370

*** Same values with or without imputed data. *Note.* With or without a self-harm history refers to hospitalisation for self-harm in the past 10 years. 'Completers' refers to those who completed the VHS in hospital following randomisation (n=248).**

To determine incremental cost per self-harm case averted non-parametric bootstrapping (1000 replications) was undertaken to test the robustness of these costs results and produce a distribution of mean costs and effects shown on a cost effectiveness plane. A cost effectiveness plane provides a way of showing the strength of the economic argument.

Appendix

Divided into four quadrants, in this particular version[5] of the cost effectiveness plane any observation in the north east quadrant means a combination of mean cost and mean effect that shows that the intervention is more costly and less effective (as future self harm cases in the VHS group are higher than in the treatment as usual group) while that in the south east quadrant is less costly but also less effective. In a similar fashion any observation in the north west quadrant indicates that the VHS is more effective but also more costly than usual care alone, whilst that in the south west quadrant indicates that it is both less costly and more effective.

Figure A1 shows the cost effectiveness plane for the intention to treat analysis including hospitalisation costs for the six participants who could not be linked to the hospital admissions database. Overall VHS **is dominant** compared to treatment as usual alone, being associated with lower costs and reduced future self-harm cases, although these differences are not statistically significant. This means that no cost effectiveness ratio is generated (as there is no incremental cost per additional self-harm case averted). The majority of the 1000 bootstraps fall in the south west quadrant, reflecting the tendency towards lower costs and better effects.

In order to further explore the uncertainty around these estimates of cost and effectiveness we looked at the potential for investment in VHS to be considered cost effective given different levels of willingness to pay by the NHS for cases of self-harm averted. While there are willingness to pay thresholds available for outcomes, such as £20,000 per quality adjusted life year gained (QALYs), there are no accepted thresholds for the outcome of self-harm cases averted. Nonetheless as Figure A2 indicates there is a 60% chance of the VHS being considered cost effective compared to treatment as usual even when the willingness to pay is zero or very low and this never falls below 50% as willingness to pay for these VHS plus treatment as usual, or treatment as usual alone increase.

These analyses have been repeated for the sub-group who completed the VHS following randomisation with similar findings. The economic case appears most promising when looking specifically at the sub-group of individuals who have a previous history of self-harm hospitalisation. Figure A3 shows the cost effectiveness plane for the self-harm history subgroup who completed the VHS. Nearly all of the bootstrapped values fall in the south west quadrant where the VHS group is less costly than treatment as usual. Figure A4 shows that there is more than a 90% probability of the intervention being cost effective regardless of willingness to pay threshold if the intervention can be targeted at those with a previous history of self-harm.

Figure A1: Cost effectiveness plane intention to treat analysis: VHS and treatment as usual versus treatment as usual only

[5] Normally the east quadrants would be more effective but in this analysis a lower value (i.e. reduction in future self-harm events is the more effective – hence the west quadrants are more effective).

Appendix

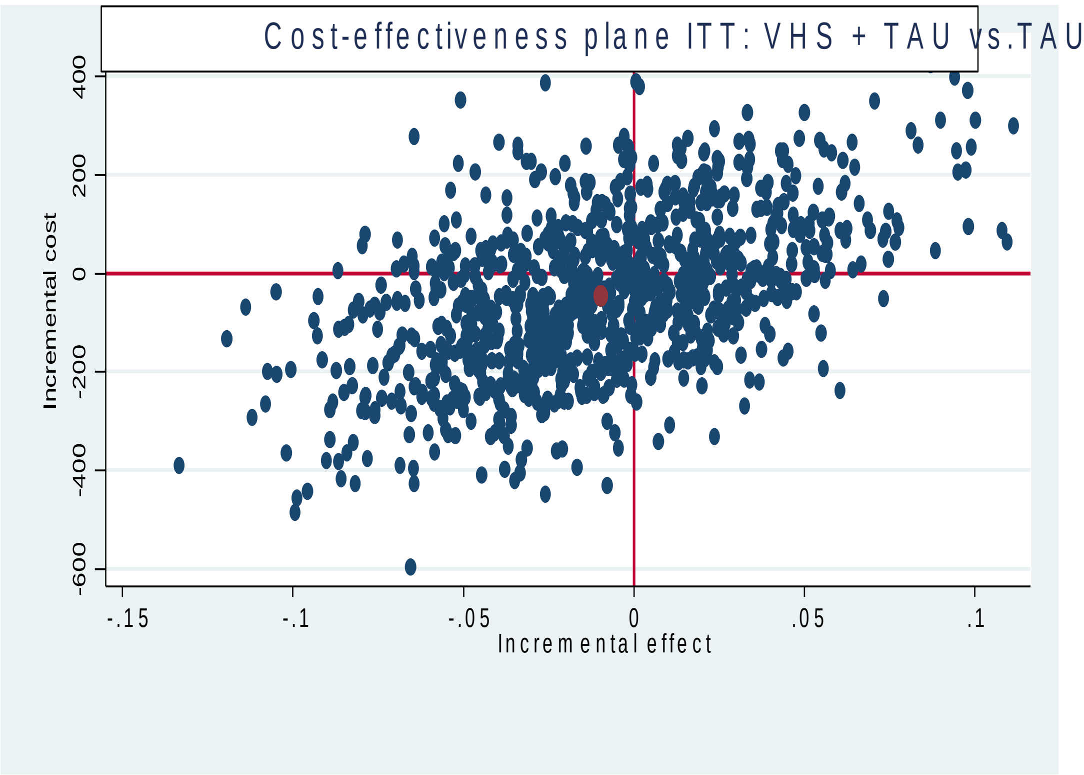

Figure A2: Cost effectiveness acceptability curve intention to treat analysis: VHS and treatment as usual versus treatment as usual only

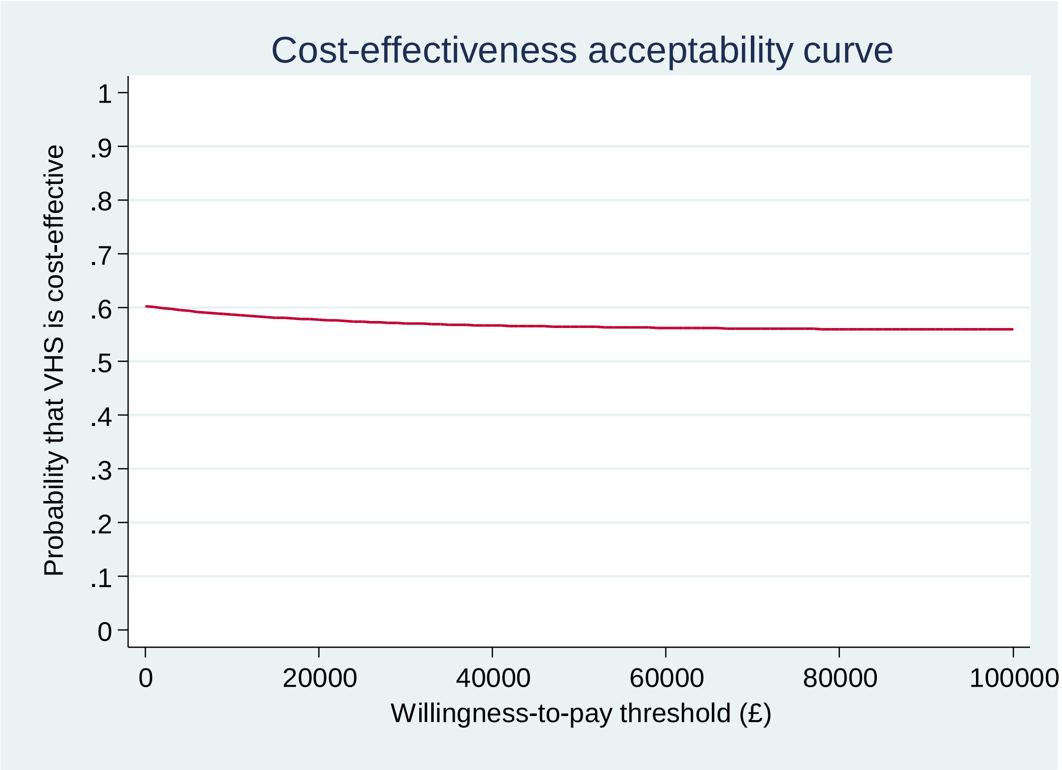

Figure A3: Cost effectiveness plane in self-harm history sub-group who completed the VHS: VHS and treatment as usual versus treatment as usual only

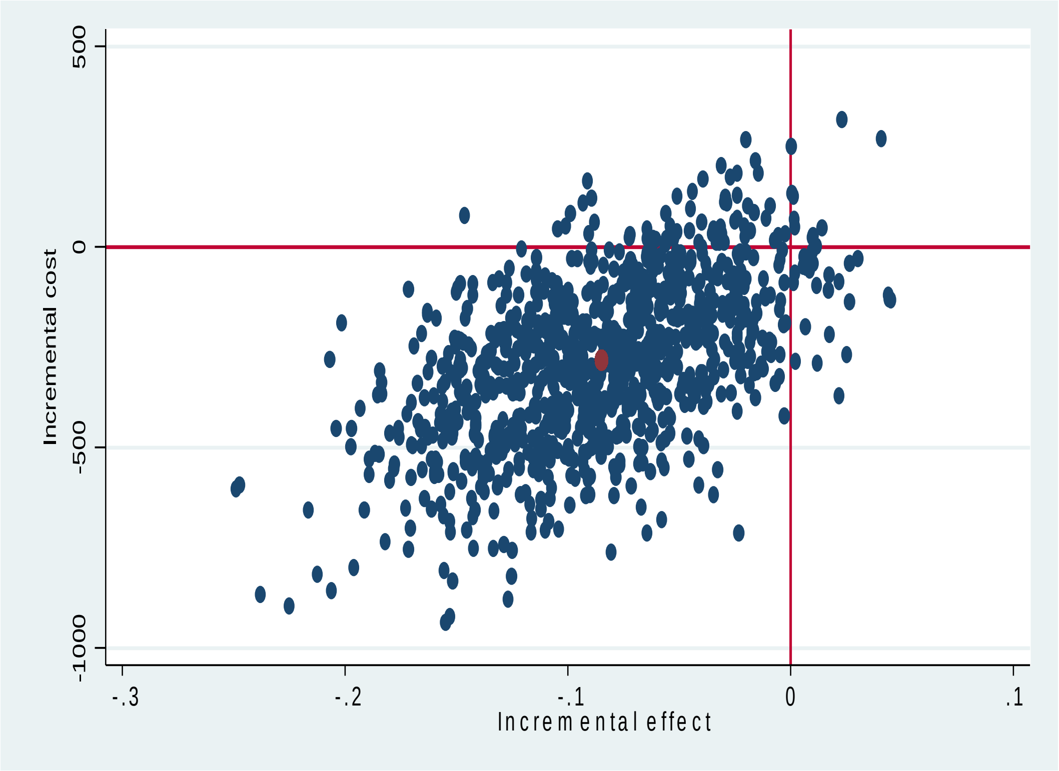

Figure A4: Cost effectiveness acceptability curve in self-harm history sub-group who completed the VHS: VHS and treatment as usual versus treatment as usual only

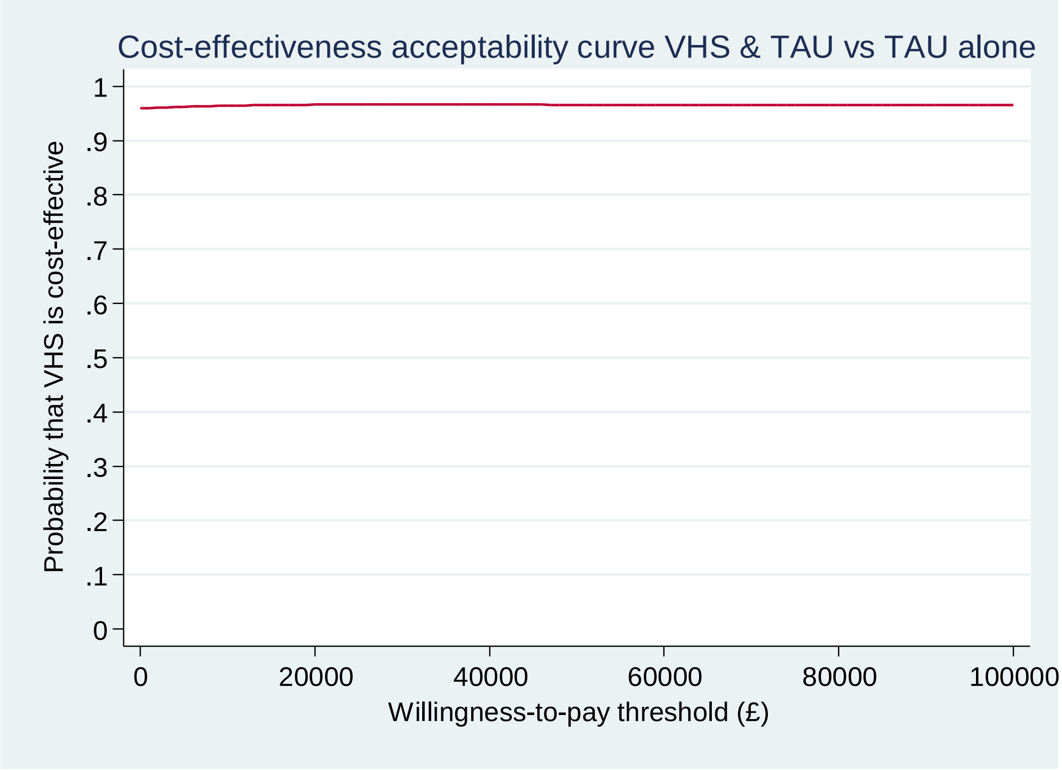

Sensitivity Analysis

Appendix

One scenario assessed the economic case for all participants in the study using observed data (listwise) and a second additionally assumed that all 6 participants who could not be linked to hospital records would be admitted to hospital at least once for self-harm (imputed). A further eight scenarios were explored, these included incremental cost per self-harm case averted using the listwise and imputed scenarios for the population who completed the VHS following randomisation (i.e., excluding the 11 individuals who did not complete the VHS intervention in hospital). Sub-group analyses were also conducted assessing the incremental cost per self-harm case averted in the intervention group versus the control group when the study population was restricted to either participants who had been hospitalised for self-harm or not in the previous ten years. The costs of delivering the VHS were also varied in sensitivity analysis from £1 to £10 without changing the conclusions of our analysis on findings. Including the cost of a psychosocial assessment for all future self-harm hospitalisations also did not alter the conclusions of the analysis.

References

1. Nixon R, Wonderling, D., & Grieve, R. . Non-parametric methods for cost effectiveness analysis: the central limit theorem and the bootstrap compared. . *Health Economics* 2010; **19**: 316-33.
2. Black WC. The CE plane: a graphic representation of cost-effectiveness. *Medical Decision Making* 1990; **10**: 212-4.
3. Fenwick E, Claxton, K., & Sculpher, M. . Representing uncertainty: the role of cost-effectiveness acceptability curves. . *Health Economics* 2001; **10**: 779-87.
